# Insecticidal effects of dsRNA targeting the *Diap1* gene in dipteran pests

**DOI:** 10.1038/s41598-017-15534-y

**Published:** 2017-11-09

**Authors:** Michelle Powell, Prashant Pyati, Min Cao, Howard Bell, John A. Gatehouse, Elaine Fitches

**Affiliations:** 1 School of Biosciences, Durham University, South Road, Durham, DH1 3LE United Kingdom; 2 Fera Science Ltd., Sand Hutton, York, YO41 1LZ United Kingdom

## Abstract

The *Drosophila melanogaster* (fruit fly) gene *Diap1* encodes a protein referred to as DIAP1 (*D* *rosophila* Inhibitor of Apoptosis Protein 1) that acts to supress apoptosis in “normal” cells in the fly. In this study we investigate the use of RNA interference (RNAi) to control two dipteran pests, *Musca domestica* and *Delia radicum*, by disrupting the control of apoptosis. Larval injections of 125–500 ng of *Diap1* dsRNA resulted in dose-dependent mortality which was shown to be attributable to down-regulation of target mRNA. Insects injected with *Diap1* dsRNA have approx. 1.5-2-fold higher levels of caspase activity than controls 24 hours post injection, providing biochemical evidence that inhibition of apoptotic activity by the *Diap1* gene product has been decreased. By contrast adults were insensitive to injected dsRNA. Oral delivery failed to induce RNAi effects and we suggest this is attributable to degradation of ingested dsRNA by intra and extracellular RNAses. Non-target effects were demonstrated via mortality and down-regulation of *Diap1* mRNA levels in *M. domestica* larvae injected with *D. radicum Diap1* dsRNA, despite the absence of 21 bp identical sequence regions in the dsRNA. Here we show that identical 15 bp regions in dsRNA are sufficient to trigger non-target RNAi effects.

## Introduction

The *Drosophila melanogaster* (fruit fly) gene *Diap1* (previously known as *thread* [*th*]) encodes a protein referred to as DIAP1 (*D* *rosophila* Inhibitor of Apoptosis Protein 1) or the “gatekeeper of death”^1,2^. This protein acts to suppress apoptosis in “normal” cells in the fly, and its elimination or loss of function results in spontaneous cell death in *Drosophila* cells and embryos^3,4^. The functional role of DIAP1 at the molecular level is to act on the apoptotic initiator caspase DRONC (*Drosophila* Nedd2-like caspase), to inhibit its activity^5^. DRONC, the homologue of the mammalian caspase-9, is required for the initiation of apoptosis through its activation of downstream caspases in a proteolytic amplification cascade^6^, and thus inhibition of DRONC activity inhibits apoptosis. DIAP1 belongs to a family of proteins referred to as IAPs (Inhibitor of Apoptosis Proteins), which share one to three tandem repeats of the Baculovirus Iap Repeat (BIR) domain (InterPro IPR001370), containing approx. 80 amino acids with a bound stabilizing zinc atom^7^. This domain is sufficient to confer anti-apoptotic activity. IAPs contain one or more BIR domains, and often contain additional domains; DIAP1 contains two BIR domains and a RING (really interesting new gene) domain (InterPro IPR001841), where the latter is a zinc-binding domain characteristic of E3 ubiquitin-protein ligases. The BIR domains and other domains in IAPs mediate protein-protein interactions that give these inhibitors their specificity^7^.

DIAP1, like other IAPs, interacts with a range of cellular components in order to regulate apoptosis^7^. Although the mechanism of action of DIAP1 is complex, down-regulating expression of its encoding gene, *Diap1*, has a simple, predictable effect on phenotype. Depletion of DIAP1 in *Drosophila* cells by RNA interference (RNAi) down-regulation of *Diap1* resulted in rapid and widespread caspase-mediated apoptosis^8,9^, and similar effects were observed in embryos expressing an RNAi construct, resulting in lethality^9^. Like DRONC, DIAP1 is present ubiquitously throughout the organism^5,10^, and at different stages of development; microarray data for transcript abundance^10,11^ show that *Diap1* expression is observed in most, if not all cells. This gene is therefore a potentially attractive target for developing insecticides based RNAi effects.

The use of double-stranded RNA (dsRNA) to down-regulate endogenous genes in insects is a well-established technique. Various studies have demonstrated the potential use of this approach as a basis for the development of target specific insecticides by delivering dsRNAs via injection, expression in transgenic plants, feeding in artificial diets, soaking or even topical application^12–14^. Following cellular uptake, dsRNAs are processed by the nuclease Dicer into 21–24 nt short interfering RNAs (siRNA) that are subsequently incorporated into the multi-subunit RNA-induced silencing complex (RISC). Catalytic argonaute proteins within the RISC complex use siRNAs as a guide to trigger complementary mRNA degradation. The production of secondary dsRNAs and transfer to other cells can lead to amplification of the silencing effect and is referred to as systemic RNA. However, RNAi effects in insects are extremely variable and dependent upon a multitude of factors including the insect species, target gene (sequence and length), mode of dsRNA delivery and stability of dsRNA to extracellular degradation^12–15^.

RNAi directed towards the *Diap1* gene has been used in previous studies to attempt to induce mortality in insects. A report that topical application or microinjection of dsRNA corresponding to the *Diap1* homologue *AeIAP1* to mosquito (*Aedes aegypti*) adults^16^ resulted in mortality has subsequently been shown to be incorrect^17,18^, although mortality was observed in cell cultures treated with *AeIAP1* dsRNA. Microinjection of dsRNA corresponding to the Diap1 homologue *LlIAP* in both adults and nymphs of the plant bug *Lygus lineolaris* caused specific down-regulation of the corresponding gene, and significantly decreased lifespan compared to controls^19^. The RNAi effect showed limited dose dependency in that injection of a minimum of 100 ng of dsRNA was required to produce mortality, but injections of greater amounts had no added effect. In addition, survival of control insects was poor in these experiments. Previous experiments have thus given inconsistent results in validating *Diap1* homologues as targets for insecticidal RNAi.

In the present paper, data are presented to show that the *Diap1* gene can be used as a target for RNAi-based insect control strategies in dipteran pests, by using data from the *Drosophila* genome as a starting point for dsRNA design. Insecticidal effects of *Diap1* dsRNAs against a model pest, housefly (*Musca domestica*) and a crop pest, cabbage root fly (*Delia radicum*) are demonstrated after injection, but not ingestion, of dsRNAs. Non-target effects were demonstrated via mortality and down-regulation of *Diap1* mRNA levels in *M. domestica* larvae injected with *D. radicum Diap1* dsRNA, despite the absence of 21 bp identical sequence regions in the dsRNA. In addition, data showing that cross-species RNAi effects based on similarity rather than identity of sequences can be effective in producing mortality is presented, suggesting that RNAi can give broader range protection, and is not necessarily invalidated by mutations in the target gene.

## Results

### Isolation of coding sequences for *Diap1* transcripts from housefly and cabbage root fly and dsRNA production

**Figure 1:**
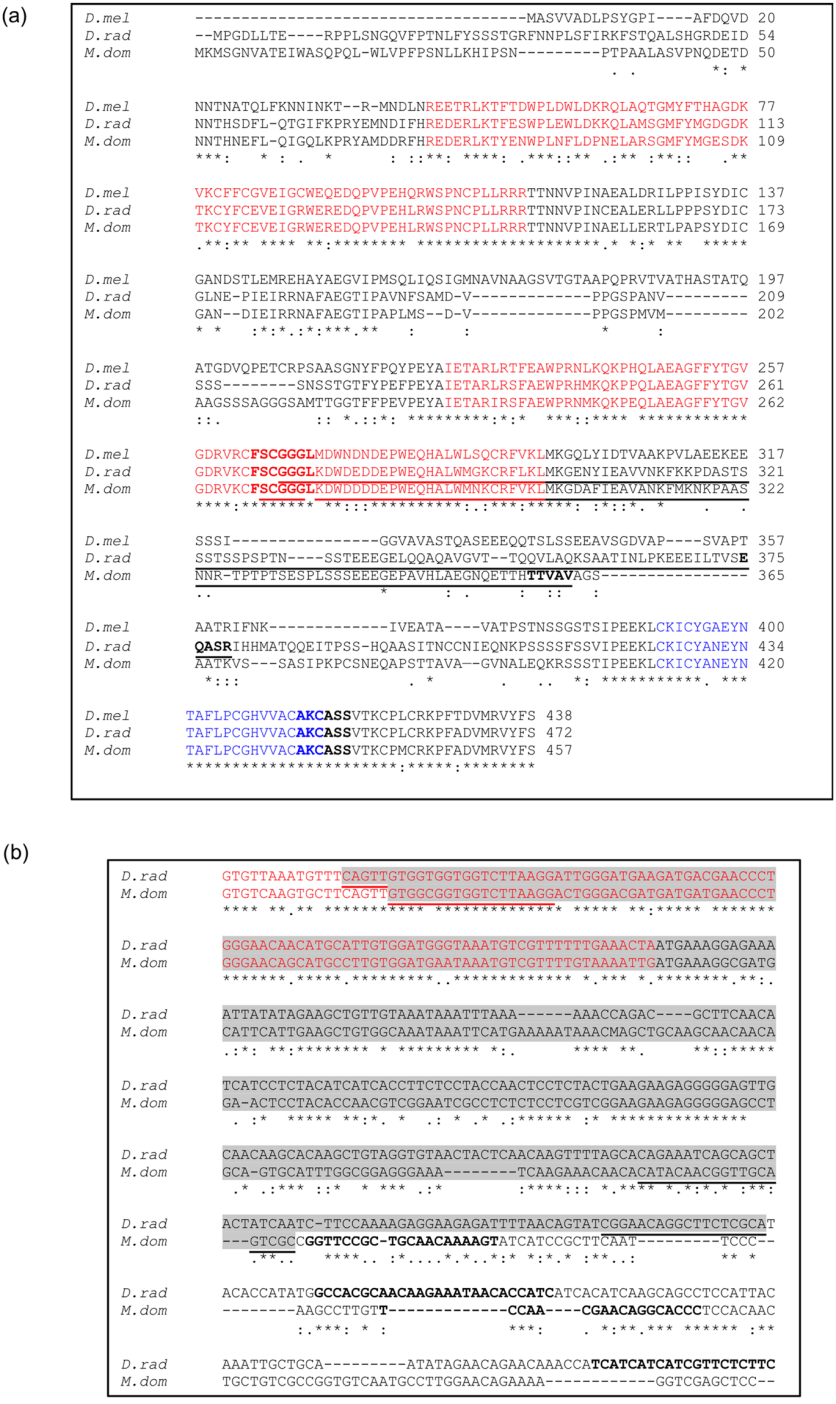
(**a**) Sequences of DIAP1 proteins predicted by *Diap1* transcripts. *D.mel* = sequence of *Drosophila melanogaster* DIAP1 protein (*th*-PA) from genome database (Accession NP-524101); *M. dom* = full-length sequence from *M. domestica* by RACE (Genbank Acc. No. MF489243). *D.rad* = full-length sequence from *D. radicum* by RACE (Genbank Acc. No. KX712114). Bold depicts conserved sequences used as a basis for degenerate PCR primers. Sequence used for dsRNA synthesis, GGGLK to TTVAV (*M. domestica*) and SCGGG to EQASR (*D. radicum*), is underlined. Blue text = RING zinc finger domain, IPR001841; and red text = BIR domain, IPR001370. (**b**) Aligned partial *Diap1* nucleotide sequences of *D. radicum* and *M. domestica* showing location of dsRNA regions (highlighted in grey) and primers used for qPCR analysis (bold text). Underlined regions indicate the position of the primers for dsRNA synthesis. Red text = BIR domain, IPR001370.

To confirm that orthologues of selected *Drosophila* gene targets were present in *M. domestica* and *D. radicum*, fragments of the coding sequences were isolated by PCR from cDNA prepared from the organisms. Sequences for the gene-specific primers were designed by comparing sequence data for orthologous coding sequences from the model dipteran *D. melanogaster*, and other well-characterised dipteran sequence databases, from *Anopheles gambiae*, *Aedes aegypti* and *Culex pipiens*. Additional information derived from a DNA sequence for an IAP orthologue present in the fly *Glossinia morsitans* was also used in the comparison. Regions of high sequence conservation were selected on the basis of amino acid sequence similarity, and alignments of corresponding nucleic acid sequence data. From these sequence comparisons degenerate primers, which could potentially be functional in a range of dipteran species were designed. PCR products from cDNA preparations were isolated and cloned. They were verified as partial sequences of orthologues of *Diap1*, encoding IAP protein inhibitors of apoptosis, by DNA sequencing and subsequent sequence analysis. Sequences were extended by 3′ and 5′ RACE to give complete coding sequences for the IAP proteins of *M. domestica* (Genbank Acc. No. MF489243) and *D. radicum* (Genbank Acc. No. KX712114). The predicted amino acid sequences of the *M. domestica* and *D. radicum* IAP proteins, with the locations of PCR primers, are shown in Fig. 1. The *M. domestica* sequence determined in this study shows 99% identity to a recently published *M. domestica* apoptosis inhibitor gene sequence (NW 004764601; XP_005178734) from a whole genome shotgun sequence (XM_011292268.1)^20^.

When amino acid sequences of the DIAP1 proteins are compared, the conserved BIR and RING domains are clearly identified, and show that all three proteins have the same domain structure (2 BIR domains followed by the RING domain, from N- to C-terminus). Sequences in the domains are well conserved between *D. melanogaster*, *M. domestica* and *D. radicum*, as are some immediately adjacent flanking regions, including the C-terminal region of the proteins. Most of the regions linking the domains, and the N-terminal regions, are highly variable both in sequence and length between the three species.

Double-stranded RNAs of approx. 300 bp were designed for the region GGGLK to TTVAV for *M. domestica* and SCGGG to EQASR for *D. radicum* (Fig. 1a). This region extended from the 3′ end of the sequence encoding second BIR domain extending to approx. half of the linker region between the second BIR domain and the RING domain in the *M. domestica* and *D. radicum* cDNAs. These dsRNAs included three regions of 13–14 nucleotides in length in BIR domains, and a region of 15 nucleotides in the variable linking region sequence that were identical in *M. domestica* and *D. radicum* cDNAs (Fig. 1b). They were produced by *in vitro* transcription, after cloning appropriate fragments into a vector containing T7 promoter sequences. Individual ssRNAs were subsequently purified and annealed to produce dsRNA. The location of the primers for dsRNA synthesis and subsequent qPCR analysis are depicted in Fig. 1b. A DNA fragment corresponding to part of the bacterial kanamycin resistance gene *nptII* was used to produce a dsRNA negative control.

### Endogenous expression of *Diap1* transcripts in *M. domestica* and *D. radicum*

**Figure 2:**
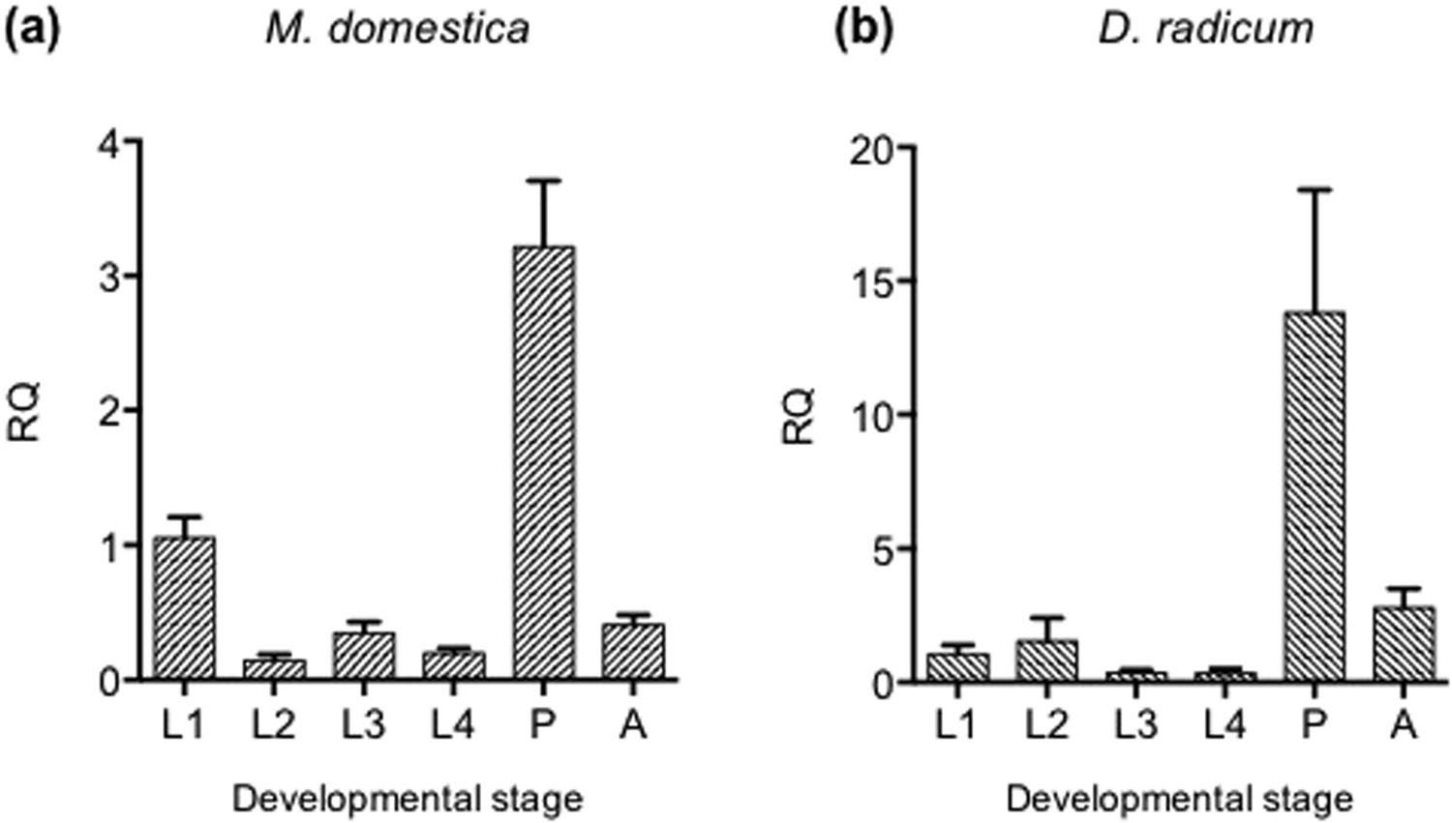
Endogenous expression of *Diap1* gene through (**a**) *M. domestica* and (**b**) *D. radicum* development, assayed by quantitative PCR. L1-L4 are different stages of larval growth, corresponding to 1^st^ instar, 1^st^ -2^nd^ instar, 2^nd^ -3^rd^ instar and 3^rd^ instar; P are pupae and A adults. Expression levels are normalised to *GAPDH* mRNA, L1 level is arbitrarily set to 1.

Real-time quantitative PCR was used to assay relative levels of *Diap1* mRNA present at different stages of *M. domestica* and *D. radicum* development. The life cycle of both species has 3 larval instars, with the length of larval development varying from 5 to 30 days depending on temperature. Under the growth conditions used (25 °C) the *M. domestica* larval stage lasts for approx. 10 days whereas for *D. radicum* the larval stage lasts for approx. 3 weeks. *M. domestica* larvae were collected every alternate day after egg hatch (day 1, day 3, day 5, day 7 and day 9; corresponding to 1st instar, 1^st^ -2^nd^ instar, 2^nd^ instar, 2^nd^ -3^rd^ instar and 3^rd^ instar pre-pupal) followed by pupae on day 10. *D. radicum* larvae were collected every 4–5 days, corresponding to *M. domestica* developmental stages, followed by pupae on day 25. Results (Fig. 2) show that expression of the *Diap1* gene was readily detectable, and could be reliably estimated at all stages of insect development. Expression was highest during pupation, when extensive tissue remodelling was taking place, but is relatively low through larval development, and in adult insects.

### Effects on mortality of injecting *Diap1* dsRNA into *M. domestica* and *D. radicum*

Results showing that *Diap1* expression was highest during pupation suggested that effects on phenotype would be most readily observed by injecting pupae, and larvae in the final (3^rd^) instar, prior to pupation, exploiting the persistent down-regulation of gene expression by dsRNA injection. Injections of dsRNA were routinely carried out on early final instar larvae, which were robust enough to be unaffected by the injection process and showed high control survival. The insects were monitored over a period of at least 6 days, to allow controls to enter pupation.

**Figure 3:**
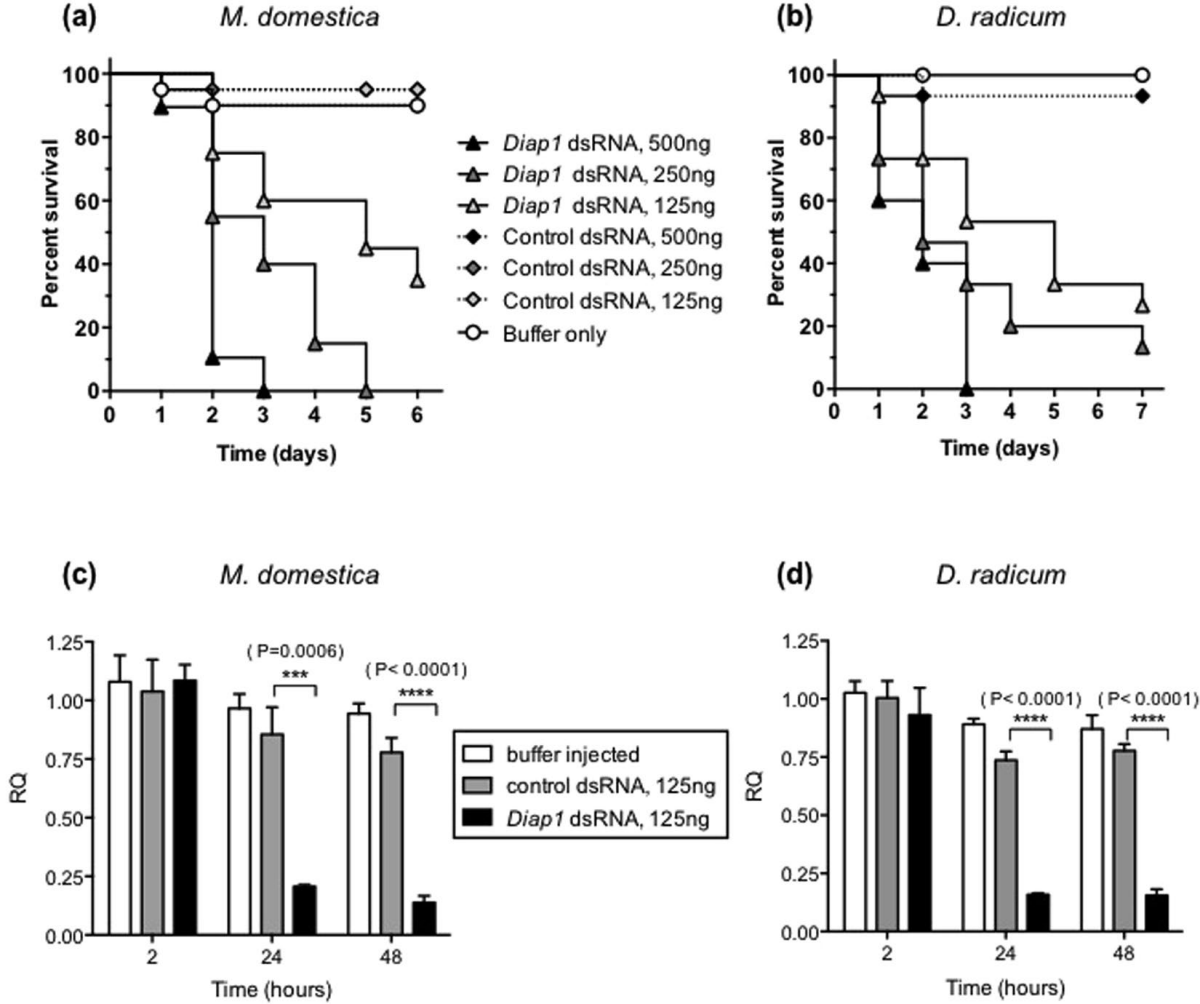
Effects of injected *Diap1* dsRNA on survival of (**a**) *M. domestica* & (**b**) *D. radicum* larvae. *nptII* dsRNA was used as a control (n = 20 per treatment). (**c**,**d**) Quantitative PCR assays showing decrease in expression of *Diap1* gene in *M. domestica* and *D. radicum* larvae injected with *Diap1* dsRNA, relative to insects injected with control dsRNA (*nptII*). Expression levels normalised to *GAPDH* mRNA. All error bars represent standard deviation of the mean of 3 independent biological replicates (5 insects per replicate). Significant differences between means (multiple t-tests) are depicted.

Results of a typical assay in which 3^rd^ instar *M. domestica* larvae were injected with known amounts of dsRNA targeting the endogenous *Diap1* gene are shown in Fig. 3a. Control survival in this assay was ≥95% for the dsRNA control, and ≥90% for the buffer control. The assay showed that dsRNA had a significant dose-dependent effect on larval mortality; a dose of 500 ng dsRNA per insect caused 100% mortality in 3 days, and a 250 ng dose caused 100% mortality in 5 days. Mortality following an injection dose of 125 ng per insect was 60% after 6 days, with surviving larvae failing to pupate. All survival curves were significantly different from controls at p < 0.0001. Injection of 2^nd^ instar *M. domestica* larvae gave similar results (not presented), with dose-dependent mortality observed after injection of *Diap1* dsRNA. The smaller size of larvae resulted in lower control survival after injection, making results less reliable.

Injection of *D. radicum* early 3^rd^ instar larvae resulted in similar dose dependent mortality (Fig. 3b) to *M. domestica* larvae. At the highest dose tested, 500 ng per insect, resulted in 100% mortality in 3 days. Doses of 250 and 125 ng per insect gave 85 and 75% mortality respectively after 7 days, showing a dose-dependent effect. Controls (buffer and dsRNA) gave mortalities of <10%. Each experimental treatment survival curve was significantly different from controls at p < 0.0001. In repeat assays <10% of larvae that survived injection of sub-lethal doses of *Diap1* (125 ng) were able to form viable puparia. These results demonstrate that RNAi effects can be translated between species if the appropriate species-specific orthologous sequence is used to prepare dsRNA.

### Down-regulation of *Diap1* expression in *M. domestica* and *D. radicum* after injection of dsRNA

Down-regulation of *Diap1* expression by injected dsRNA (250 ng) was demonstrated in a time course experiment using 3^rd^ instar larvae (Fig. 3c and d). No effect on *Diap1* mRNA levels was observed after 2 hours, comparing injection of dsRNA targeting the gene to control dsRNA. After 24 hours, mean *Diap1* mRNA levels showed a significant reduction to <30% of the level in insects injected with control dsRNA, at all injection doses of *Diap1* dsRNA (100–500 ng dsRNA per larva; 125 ng results presented in Fig. 3c and d). There was no obvious dose-dependency in the decrease of *Diap1* mRNA observed (results not shown). Mean *Diap1* mRNAs levels in treated insects were also significantly lower as compared to controls 48 hours after injection, but again no obvious dose-dependency was observed. These data show that injection of dsRNA targeting the *Diap1* gene gives effective, persistent down-regulation of gene expression.

### Phenotypic effects of *Diap1* dsRNA in *M. domestica* and *D. radicum* after injection of dsRNA

**Figure 4:**
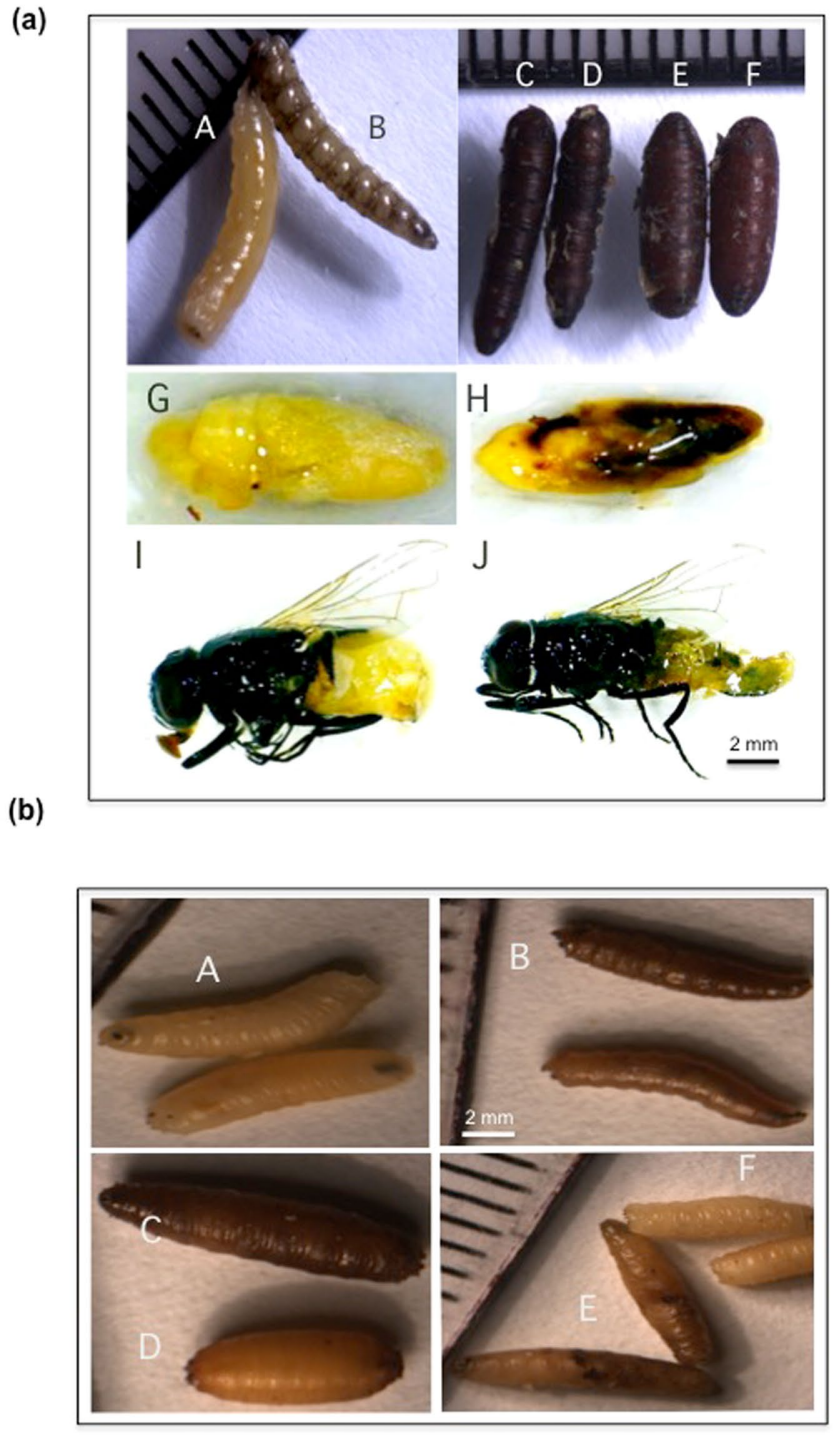
(**a**) Phenotypic effects of down-regulation of *Diap1* by dsRNA injection into *M. domestica*. Control insects were injected with *nptII* dsRNA. A and B: Pre-pupae injected as 3^rd^ instar larvae with (A) control and (B) *Diap1* dsRNA (300 ng/larva); C-F: Pupae injected as pre-pupal larvae with (C & D) *Diap1* dsRNA and (E & F) control dsRNA (300 ng/larva); G and H: Dissected pupae injected as pre-pupal larvae with (G) control and (H) *Diap1* dsRNA (500 ng/larva); I and J: Emerged adults injected as pre-pupal larva with (I) control and (J) *Diap1* dsRNA, (500 ng/larva). (**b**) Phenotypic effects of down-regulation of *Diap1* by dsRNA injection into *D. radicum*. Control insects were injected with *nptII* dsRNA. A and B: Pre-pupal larvae injected with (A) control dsRNA and (B) *Diap1* dsRNA (300 ng/larva); C and D: Pupae injected with (C) *Diap1* dsRNA and (D) control dsRNA (300 ng dsRNA/pupa); E and F: Pupae injected as larvae with (E) *Diap1* dsRNA (apoptotic patches evident) and (F) control dsRNA (200 ng dsRNA/larva).

Phenotypic effects following injections of 3^rd^ instar or pre-pupal (late 3^rd^ instar) larvae with *Diap1* dsRNA were similar for *M. domestica* and *D. radicum;* insects remained in a larviform state even though darkening of the cuticle took place, and those that reached a pupal-like state were malformed as compared to controls (Fig. 4a and b, insects A-F). This suggests that excess apoptosis was occurring, leading to necrosis and an immune response. *M. domestica* injected at the pre-pupal stage were less sensitive to *Diap1* dsRNA and a higher dose (500 ng dsRNA) could be administered without causing high levels of mortality. The persistence of dsRNA effects was shown by the observed phenotype of developing adult insects following the injection of pre-pupal larvae with *Diap1* dsRNA. As shown in Fig. 4a (G-J) tissue blackening, frequently associated with the ovaries and abdominal regions, was commonly observed in developing adults that had been injected just before pupation.

### Demonstration of increased apoptopic activity in insects injected with Diap1 dsRNA

**Figure 5:**
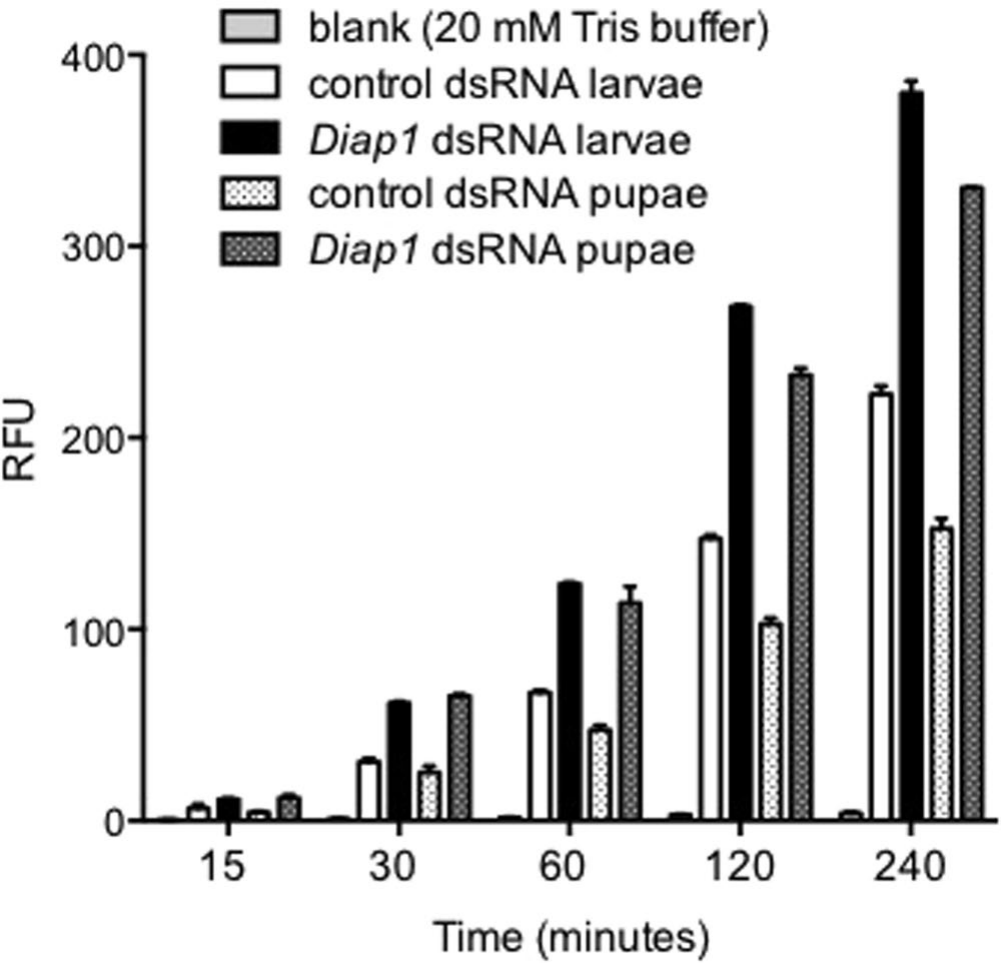
Biochemical evidence for decreased inhibition of apoptotic activity by the *Diap1* gene. Detection of caspase activity in *M*. *domestica* larvae and pupae injected with *Diap1* dsRNA and control dsRNA (*nptII* dsRNA). Caspase 3/7 activity was detected by an increase in relative fluorescence units (RFU) shown by the substrate at different time intervals from 15 to 240 minutes.

The disruption of endogenous apoptosis regulation was visualised directly at the biochemical level by assaying the activity of the apoptopic proteases caspases 3/7 in insects injected with *Diap1* or control dsRNA. *M. domestica* larvae and pupae were injected with a sub-lethal amount (150 ng/insect) of *Diap1* dsRNA, or the same amount of control dsRNA. Insects injected with *Diap1* dsRNA had approx. 1.5-2-fold higher levels of caspase activity than controls 24 hours post injection, showing directly that inhibition of apoptotic activity by the *Diap1* gene product had been decreased (Fig. 5).

### RNAi effects in *M. domestica* adults

Newly emerged housefly adults were injected with 150 and 500 ng *Diap1* dsRNA and survival was monitored for 6 days. No indication of any mortality resulting from injection with *Diap1* dsRNA was observed at either dose (results not shown). However, the failure to observe a phenotype resulting from an RNAi effect could be a result of low gene expression at this developmental stage, suggesting it is not essential for survival.

To investigate if injection of dsRNA caused down-regulation of gene expression in adults, injected flies were collected after 48 and 72 hours, and used to prepare cDNA for analysis of *Diap1* transcript levels by qPCR. In agreement with survival data, no significant down-regulation of *Diap1* transcript was observed after injecting *Diap1* dsRNA at either dose or either time point (results not shown). These results indicate that the RNAi effect induced by dsRNA injection was dependent on developmental stage in housefly, with larvae showing a robust effect, which was absent in adults.

### Effects of injection of *D. radicum Diap1* dsRNA on *M. domestica* larvae

**Figure 6:**
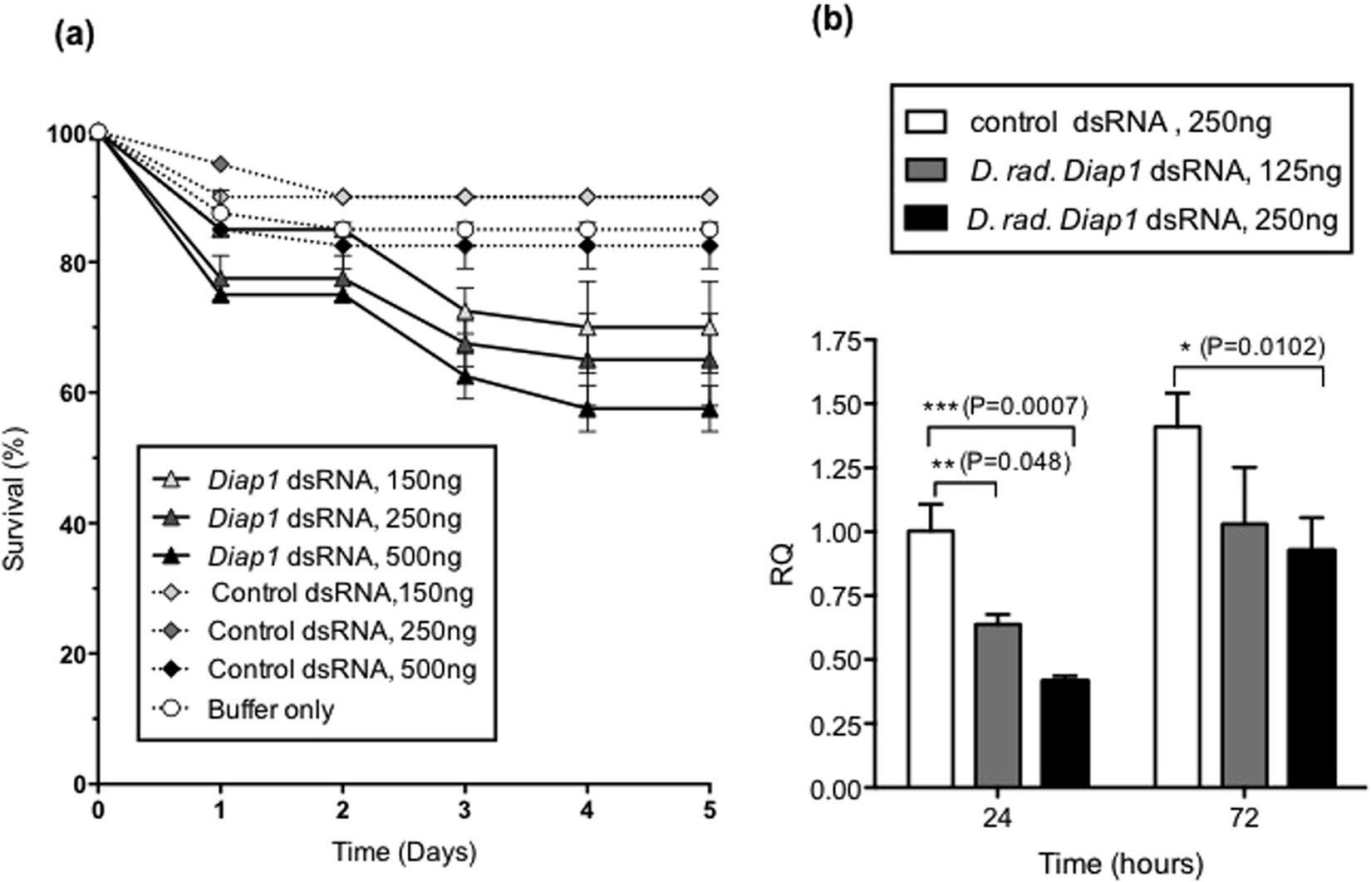
Cross-species effects of *Diap1* dsRNA (**a**) Survival of 3^rd^ instar larvae of *M. domestica* after injection of different doses of *D. radicum Diap1* dsRNA. *nptII* dsRNA was used as a control (n = 20 per treatment). (**b**) Quantitative PCR assays showing decrease in expression of *Diap1* gene in *M. domestica* injected with *D. radicum Diap1* dsRNA, relative to insects injected with control dsRNA (*nptII*). Expression levels normalised to *GAPDH* mRNA. Error bars represent standard deviation of the mean of 3 technical replicates (5 insects per replicate). Significant differences between means (multiple t-tests) are depicted.

To investigate whether effects of dsRNA on gene expression can be produced using gene sequences of related species, 3^rd^ instar *M. domestica* larvae were injected with *D. radicum Diap1* dsRNA (Fig. 6). Larval survival was reduced in insects injected with 150–500 ng dsRNA but the mortality was not as high as in assays using dsRNA corresponding to the species from which the sequences were cloned (Fig. 6a) with 55, 60 and 65% survival recorded for injection doses of 500, 250 and 125 ng, respectively. Survival curves for 500 and 250 ng, but not the 150 ng dose, were significantly different from control treatments at p < 0.05. Mean transcript levels analysed by qPCR were significantly reduced 24 hours after injection of 125 and 250 ng *Diap1* dsRNA and were 40 and 60%, respectively lower than the control treatment. Although significant levels of down-regulation were observed for *M. domestica* larvae injected with Diap1 dsRNA the degree of down regulation was not as great as that observed in *M. domestica* assays using *M. domestica Diap1* dsRNA; these results are in agreement with injection assay data. Furthermore, some recovery of mRNA levels was observed 72 hours post injection in the cross species assays and mean mRNA levels were only significantly reduced for insects injected with the higher dose of 250 ng *Diap1* dsRNA. This suggests that whilst cross-reactivity of dsRNA can result in reduced target mRNA levels, the effects may be less persistent due to the use of dsRNAs containing sequences with limited homology to endogenous mRNAs, ultimately resulting in lower levels of mortality. Significant down-regulation of the *M. domestica Diap1* transcript was also observed in insects injected with 500 ng *D. radicum Diap1* dsRNA (results not shown).

### Oral delivery of dsRNA to *D. radicum*

**Figure 7:**
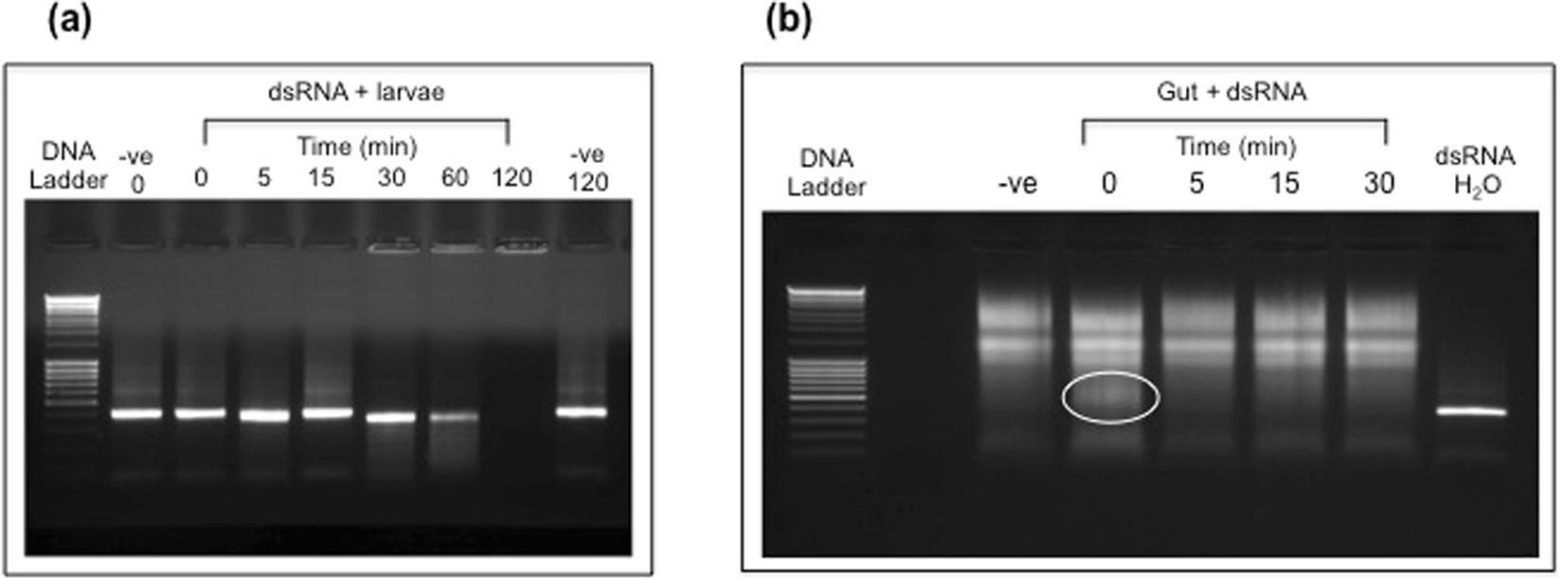
Stability of dsRNA in the presence of *D. radicum* larvae. (**a**) *In vivo*; larvae were immersed in solutions containing dsRNA and samples were taken at specified time points and separated on a 1.2% agarose gel. Negative controls show dsRNA alone (i.e. no larvae present at time 0 and 120 mins). (**b**) *In vitro* stability of dsRNA in *D. radicum* larval gut; dsRNA was incubated in gut extracts and samples taken at the indicated time points. 10 µl of extract (approx. 1 gut equivalent) loaded in all lanes. Negative control is gut sample alone and positive control is dsRNA in distilled water. Circle indicates presence of intact dsRNA at time 0.

Initially, attempts to administer dsRNA orally to *D. radicum* larvae were made by using lyophilised swede discs re-hydrated with dsRNA in sterile water. However, analysis by gel electrophoresis demonstrated that degradation of the dsRNA occurred within 5 minutes of addition to the swede discs (results not shown) and thus none was delivered to larvae. Delivery was also attempted by soaking larvae in solutions containing dsRNA. Solutions were analysed by gel electrophoresis after different times of exposure to larvae. The dsRNA was found to be significantly degraded after an incubation period of 60 minutes, as evidenced in Fig. 7a by a reduction in intensity of the dsRNA fragment on agarose gels, and was undetectable after 2 hours exposure. This was indicative of nuclease activity in the regurgitant or excreta from guts of the larvae. Nevertheless, survival of dsRNA for approx. 60 minutes was considered as possibly sufficient to enable ingestion and uptake by the larvae. In repeated assays no evidence for effects of dsRNA on survival, phenotype or down-regulation of *Diap1* expression were observed even when larvae were immersed for up to 18 hours in solutions containing dsRNAs.

When dsRNA was exposed to a soluble protein extract from homogenised larval guts *in vitro*, rapid degradation of dsRNA was observed. Results are shown in Fig. 7b and demonstrate that no intact dsRNA was present after 5 minutes incubation. The use of gut homogenate rather than gut contents gave an indication of dsRNA susceptibility to degradation by intracellular and extracellular nuclease activity. Nevertheless, this result combined with the observed degradation of dsRNA in the presence of larvae (i.e. extracellular nuclease activity) suggests that the failure to observe any effect from orally administered dsRNA was due to susceptibility to degradation.

## Discussion

Cloned sequences corresponding to transcripts of homologues of the *Drosophila Diap1* gene from two related dipteran species, housefly (*M. domestica*) and cabbage root fly (*D. radicum*) showed the expected pattern of sequence conservation between regions corresponding to functional domains in the IAP proteins, and variability in regions corresponding to the “linkers” joining these domains together. In theory, the differences in sequence identity between conserved and non-conserved regions of protein products of target genes should allow the design of dsRNAs which are highly specific towards a single target (using variable regions) or which could potentially show activity towards orthologous genes in related species, or paralogous genes in the same species. The paper by Baum *et al*.^21^, where dsRNA was delivered by transgenic plants to related coleopteran species, demonstrated that cross-species effects were possible. However, three identical regions of 20–29 nucleotides can be identified in the sequence alignment published by Baum *et al*.^21^, providing an explanation for observed non-target effects. Nevertheless the present study is in agreement with the earlier work, in that dsRNA directed against a target gene in one species can have an RNAi effect when administered to a related species, although the effect is quantitatively less when similar doses of dsRNA are compared. The dsRNAs targeting IAP-coding mRNAs in the present study included both conserved and variable sequence sections, although they were carefully selected from the same transcript region to be directly comparable for both dipteran species.

The mechanism through which an RNAi effect is produced requires the production of fragments of siRNA from dsRNA, by the enzyme Dicer. The dsRNA fragments have a 19–21 bp core of dsRNA, with 2 base ssRNA extensions on the 3′ end of each strand. These siRNAs are loaded into the RISC complex, followed by degradation of one strand; the remaining strand then base pairs to the target mRNA, directing its degradation. The conventional view is that a perfect sequence complementarity with the target mRNA over the 21–24 base ssRNA strand is required to result in RNA degradation. Bolognesi *et al*.^22^ demonstrated that a single 21 bp match within a 240 bp fragment was sufficient to induce RNAi effects in the susceptible coleopteran *Diabrotica virgifera virgifera*, although a 21 bp short interfering (si) RNA was not. In a comprehensive study involving insect species representing 10 families and 4 orders to investigate the potential for cross-species effects, Bachman *et al*.^23^ demonstrated that a shared sequence length of ≥21 nt was required to induce phenotypic RNAi effects in feeding assays. It is therefore surprising that the maximum continuous stretch of base identity in the region selected for dsRNA production between *M. domestica* and *D. radicum* was 15 bases, and yet reasonably good cross-species RNAi effects were observed, including down-regulation of transcripts. A similar mortality phenotype could have resulted from miRNA-like effects on translation of the IAP-coding mRNA, which would not require wholly accurate base matching, but a 10-fold decrease in transcript abundance would not be expected. Unfortunately the study by Bachman *et al*.^23^ did not include any Dipteran species and this was because, as we observed in this study, they are often refractory to dietary dsRNA. Our result suggests that the RNAi machinery in *Musca domestica* may be more tolerant of sequence mismatches than was expected and further work to explore this phenomenon would be useful in view of potential uses of dsRNAs as insecticidal molecules. Toleration of sequence mismatches will improve the chances of dsRNAs being effective against related pest species, while still maintaining specificity in avoiding effects on beneficial insects. It will also decrease the possibility of evolution of resistance, since single base mutations in the target gene will be unlikely to be effective in preventing RNAi.

The expression profile for the *Diap1* gene over insect development, with expression detectable at all stages but increased at pupation, is consistent with previous data for other dipterans, including *Drosophila* (Flybase) and mosquitoes^22^. Apoptosis is clearly required during tissue remodelling on pupation, but is also involved in immune responses and other responses to stress; a capacity for apoptosis over the entire life cycle of the organism requires the presence of IAPs to regulate the activity of apoptotic proteases. The biochemical data presented here, showing an increase in apoptotic protease activity in fly larvae treated with dsRNA targeting the IAP-encoding gene, demonstrates that *Diap1* does play an important role in preventing unwanted apoptosis. Malformation of pupae and adults as a result of injecting *Diap1* dsRNA into pre-pupal larvae, or pupae, demonstrates that failure to control apoptosis properly leads to aberrant metamorphosis, even when doses are sub-lethal. Further work would be required to determine how interference with control of apoptosis leads to the phenotypes that are observed.

Down-regulation of *Diap1* gene expression in dipterans caused by microinjection of dsRNA targeting the gene in *A. aegypti* was investigated by Puglise *et al*.^17^. In these experiments, which were carried out on adult insects, 1000 ng per insect of an injected dsRNA of 555 bp caused approx. 30% reduction in *AeIAP1* transcript level 2 days post injection. No effect was seen after 3 days, or with other *Diap1* dsRNA sequences. Unsurprisingly, this comparatively minor effect on gene expression did not result in increased mortality in insects injected with *AeIAP1* dsRNA. The results reported here are in strong contrast. Injection of 500 ng per insect of dsRNA directed against *M. domestica* or *D. radicum Diap1* genes resulted in 100% mortality of larvae, under conditions where injected control survival was >90%. Mortality was dose-dependent, with only 30% survival recorded at a dose of 125 ng of dsRNA per insect, and even at this lower dose, *Diap1* gene expression was down-regulated to <20% of control transcript levels. The dipteran species used in this paper are significantly larger than mosquitoes. However, the apparent discrepancy between the two sets of results is explained by further experiments carried out as part of the present project. When adult *M. domestica* flies were injected with up to 500 ng per insect of dsRNAs directed against *Diap1*, no mortality was observed over a 6 day assay period, and no significant down-regulation of *Diap1* transcripts could be detected. Our results confirm the observations of Puglise *et al*.^17^ that adult dipteran insects are insensitive to injection of IAP dsRNA, but are novel in showing that larval and pupal insects are sensitive. This change in sensitivity to injected dsRNA with development is important in developing RNAi as an insecticidal strategy. Understanding the mechanism(s) responsible could lead to increased efficacy for dsRNAs as insecticides, but will require further experimentation.

In agreement with the conclusions of Puglise *et al*.^17^ who reconsidered earlier reports of external administration of dsRNA causing RNAi effects in *A. aegypti*, the present work found no evidence for oral administration of dsRNA causing down-regulation of target genes in dipterans The incorporation of dsRNAs in diet or delivery via feeding on transgenic plants expressing dsRNAs has been shown to cause RNAi effects in coleopteran, lepidopteran, hemipteran, blattodea and dipteran insect species^21–36^. However, effects of orally administered dsRNAs are highly variable and often negligible or absent. The reason(s) for this lack of effect have yet to be fully elucidated, but stability of the dsRNA to degradation prior to uptake into the cells where the RNAi effect will be produced is clearly involved. Our own observations show that dsRNA is stable over a time period of days when in solution in buffers at room temperature, and is not readily degraded by non-specific nucleases, but the results presented here and elsewhere suggest that insects contain enzymes capable of rapidly degrading dsRNA^37–40^.

Evidence for the involvement of RNAses in the digestion of orally delivered dsRNA by the desert locust *Schistocerca gregaria* was reported by Wynant *et al*.^39^. Hemipterans, such as plant bugs, appear to contain dsRNases in their saliva^38^, and this may be a general phenomenon, although degradative enzymes produced in the gut may also be involved.

Exploitation of RNAi effects to protect crops against insects has yet to be introduced in commercial agriculture, although development of transgenic plant products has been taking place for at least 10 years. Cabbage root fly, where larvae feed on an inaccessible part of the plant, is the type of pest that would be ideally targeted by this strategy; the results presented here demonstrate that crop protection using dsRNA targeting the *Diap1* gene would be feasible, but improved delivery methods will need to be developed to improve efficacy.

## Materials and Methods

### Insects

*Delia radicum* cultures were maintained as described by Finch and Coaker^41^. All developmental stages were maintained at 20 °C, 16 hours light: 8 hours dark and 65% relative humidity. The larvae were fed on organic swede. Adults maintained in Bugdorm-1 tents (Megaview Science Education Services Co., Ltd) were fed using cotton wool swabs soaked with solutions of sugar (10% [w/v]), yeast extract (1.25% [w/v]) and dried milk powder. *Musa domestica* culturing was carried out under the same environmental conditions as for *D. radicum*. Adults were fed as described for *D. radicum* and larvae were fed on a diet consisting of organic bran, grassmeal, yeast, malt and milk powder (40 + 20 + 10 + 3 + 3 by weight) made up to a paste with water^42^.

### Isolation of *Diap1* transcript sequences from *M. domestica* and *D. radicum*

Total RNA was prepared from insects using Tri reagent (Sigma Chemical Co.) according to the manufacturer’s protocol. Total RNA (1 µg) was treated with Turbo DNAse (Ambion) to remove traces of genomic DNA. Samples were then treated with phenol:choloroform:isoamyl alcohol and RNA was precipitated using ethanol. RNA pellets were re-suspended in nuclease free water and first-strand cDNA was synthesised from 1 μg of total RNA using a High Fidelity Reverse Transcription kit (Roche) with 500 ng oligo(dT)18 primer as described in the protocol supplied. As shown in Supplementary Table S1 (Sigma Chemical Co.) degenerate primers were designed to amplify a PCR product of 470 bp of *Diap1* from *M. domestica* and *D. radicum*. PCR reactions were performed using *Taq* DNA polymerase (Fermentas, Life Technologies) under the following PCR conditions: 94 °C for 5 minutes (1 cycle), 94 °C for 30 seconds, 58 °C for 30 seconds, 72 °C for 30 seconds (30 cycles) and 72 °C for 7 minutes (1 cycle).

The PCR products were cloned into pJET1.2 (CloneJET PCR Cloning kit, Thermo Scientific Life Science Research) as described in the manufacturer’s protocol. The identity of the resulting DNA fragments was confirmed by DNA sequencing.

Based on partial coding sequences obtained, gene specific primers (Supplementary Table S1) were designed. RACE (Rapid Amplification of cDNA Ends)-ready cDNA was prepared from 1 µg of total RNA extracted from whole larvae using a SMART̂2 RACE cDNA Amplification Kit (Clontech) and RACE (3′ and 5′) was performed as described in the manufacturer’s instructions. Both RACE products were introduced into pCR2.1 vector and sequenced. RACE product sequences were aligned using Sequencher software (Gene Codes Corp.) to give a complete cDNA sequence. Multiple clones were sequenced to check for amplification errors.

### Quantitative Real-Time PCR

Quantitative PCR (qPCR) was performed on *M. domestica* and *D. radicum* cDNA using an ABI StepOne instrument (Thermo Fisher Scientific) with ΔΔCT methodology. In all cases 3 biological replicates containing 5 pooled insects for each target gene and time point were analysed, except for endogenous gene expression experiments. Primers were designed using Primer express software for real-time PCR v 2 (Applied Biosystems) (Supplementary Table S1). Reaction mixtures (20 μl) contained 1x SYBR® Green JumpStart™ *Taq* ReadyMix™ (Sigma Aldrich), ROX as a reference dye, 20 μM PCR primers and 200 ng of cDNA or water as a negative control. Reactions were run in triplicate and analysis of amplification profiles was carried out using StepOne software (Thermo Fisher Scientific). The qPCR assays were conducted according to MIQE (Minimum Information for Publication of Quantitative Real-Time PCR Experiments) guidelines. *GAPDH* was used as the reference gene for normalisation of *Diap1* gene expression. Following qPCR the obtained RQ data was plotted using GraphPad Prism 6 software.

### *In vitro* dsRNA Synthesis

Target templates for *in vitro* transcription were generated using gene specific primers based on cloned *M. domestica and D. radicum Diap1* coding sequences (Supplementary Table S1). PCR was conducted using Phusion® High-Fidelity DNA Polymerase (Fermentas, Life Technologies) under previously stated conditions, with the exception that amplification cycles were reduced to 15. Products of 277 bp from GGGLK to HTTVAV (*M. domestica*) and 333 bp from SCGGG to EQASR (*D. radicum*) (Fig. 1) were restricted with *XhoI* and *XbaI*, ligated into plasmid Litmus28i (New England BioLabs) and purified plasmids were verified by DNA sequencing. *M. domestica and D. radicum Diap1* dsRNAs were prepared using Megascript T7 transcription kit (Ambion), according to the manufacturer’s instructions. For control treatments dsRNA was prepared corresponding to a region of a bacterial nptII resistance gene (*nptII*). T7-RNA polymerase was used in transcription reactions, with target template linearized with *XhoI* and *XbaI* to generate single-stranded RNA (ssRNA). Each ssRNA was precipitated by adding equal amounts of lithium chloride and nuclease-free water and re-suspended in 50 μl 20 mM Tris buffer, pH 7.0. Aliquots of ssRNA were analysed by agarose gel electrophoresis to check the integrity of samples. Finally, equal amounts of ssRNA were added together and annealed by heating the reaction to 80 °C and allowing it to cool to room temperature overnight.

The resulting dsRNAs were quantified using spectrophotometry and precipitated, as described earlier, for concentration of samples. The dsRNA was re-suspended in 20 mM Tris buffer, pH 7.0 and analysed by agarose gel electrophoresis to confirm that the size of product was similar to the DNA insert in the pLitmus construct. Typically, the system yielded 60–100 μg of dsRNA for an input of 1 μg of dsDNA template.

### Injection Bioassays

*Musca domestica* and *D. radicum* were chilled on ice for 10 to 15 minutes prior to injection. Larvae, pupae and adults were injected in the abdomen on the ventral side with 125–500 ng dsRNA using pulled glass needles and a Nanoject II^TM^ injector (Drummond Scientific Company). Initial assays were based on observations of subsequent mortality of insects, over a period up to 7 days, comparing insects injected with dsRNAs directed towards target gene (*Diap1)* with insects injected with control *nptII* dsRNA. Subsequent assays investigated a range of RNAi-induced effects, depending on the developmental stage of the insects assayed.

To investigate if *Diap1* derived from *D. radicum* influenced *Diap1* gene regulation in *M. domestica*, 3^rd^ instar larvae of *M. domestica* were injected with *D. radicum Diap1* dsRNA (*nptII* dsRNA as control) at doses of 125–500 ng per larva. Injected larvae were frozen 24 and 72 hours after injection and prepared for qPCR as described previously.

### Caspase 3/7 Assay

*Musca domestica* larvae and pupae were injected with a sub-lethal amount (150 ng/insect) of *Diap1* dsRNA in 20 mM Tris buffer, control treatments were *nptII* dsRNA or buffer alone. Total protein was extracted from larvae 24 hours post injection; 50 mg of pooled tissue (from 10 insects) was homogenized in 300 µl 1XPBS and incubated at 4 °C for 15 minutes. The homogenate was centrifuged and the supernatant was used for quantification of total protein by BCA method (Pierce) using BSA as standard. Two μg of total protein was used in a 100 μl assay reaction. The caspase 3/7 assay was performed using a Promega ApoOne kit which uses a flourometric substrate (Rhodamine 110, bis-[N-CBZ- L-aspartyl-L-glutamyl-L-valyl-L-aspartic acid amide]; Z-DEVD-R110) following the manufacturer’s protocols. Relative fluorescence unit (RFU) released by the substrate in response to increase in caspase activity with time was measured.

### Oral delivery of dsRNA to *D. radicum*

Preliminary experiments established that larvae would survive when immersed in water for more than 18 hours and that red food dye included in the dipping solution could be detected in larval guts indicating that the larvae had ingested some of the surrounding solution. The stability of dsRNA in the absence and presence of larvae was assessed by analysing samples on 1.2% agarose gels taken at different time points after immersion. Third instar *D. radicum* larvae were subsequently immersed in dsRNA containing solutions (100 ng/µl in sterile water) for 18 hours (fresh dsRNA solution provided every 2 hrs during the day), thereafter two larvae were placed in a petri dish containing a swede disc (15 replicates per treatment) and then placed individually in petri dishes (3 cm diam.) sealed with parafilm. Survival until pupation and adult emergence was monitored.

### *In vitro* dsRNA degradation assay

It is not possible to separate gut contents from the gut tissue of *D. radicum* larvae and thus gut samples (including contents) were dissected from 10 3^rd^ instar larvae and homogenised in 100 µl of Ringers solution (125 mM NaCl, 1.5 mM CaCl_2_, 5 mM KCl pH 7.31). Samples were then centrifuged (13 000 rpm 7 mins; room temperature) and 10 µl (approx. 1 gut equivalent) of the resultant supernatant incubated with 500 ng of dsRNA for 0–60 minutes. The integrity of dsRNA was assessed by gel electrophoresis.

### Statistical analysis

Data were analysed using Prism 6.0 (GraphPad Software Inc.). Survival data were analysed using survival analysis (Log-rank Mantel-Cox test). Mean relative mRNA levels were analysed by student *t-tests*. Analysis of caspase activity was conducted using ANOVA analysis (with Bonferroni-Dunn post-hoc tests) to determine any significant differences between treatments. The accepted level of significance was *P* < 0.05 in all cases.

### Data availability

The datasets generated during and/or analysed during the current study are available from the corresponding author on reasonable request.

## Electronic supplementary material

Primers sequences
