# Supplementary material for: Insecticidal effects of dsRNA targeting the Diap1 gene in dipteran pests
Source: Sci Rep. 2017 Nov 9;7:15147. doi: 10.1038/s41598-017-15534-y (PMC5680328; doi:10.1038/s41598-017-15534-y)
Supplement: Supplementary file 1 — Primers sequences [file 41598_2017_15534_MOESM1_ESM.pdf]

Title: Insecticidal effects of dsRNA targeting the *Diap1* gene in dipteran pests

Authors: Michelle Powell, Prashant Pyati, Min Cao, Howard Bell, John A. Gatehouse and Elaine Fitches.

Supplementary Table S1. Sequence of forward (Fwd)/ reverse (Rev) primers used for cloning of *Diap1* sequences from *M. domestica* and *D. radicum*, dsRNA synthesis and qPCR analysis.

Primer	Sequence 5'-3'
Degenerate *Diap1* RACE	
Fwd	GYTTCAGYTGYGGBGGWGGBY
Rev	BACVGANGADGCGCAYTTGGC
Gene Specific *Diap1* RACE	
M. domestica	
3' RACE	GCTGCGGGGGTGGTCTTAAGGACTGGGACG
5' RACE	CGGACGAGGCGCATTTGGCACAGGCCAC
D. radicum	
3' RACE	ACAGTATCGGAACAGGCTTCTCGCATACACC
5' RACE	ACCTACAGCTTGTGCTTGTTGCAACTCCCCCTC
qPCR- GAPDH	
M. domestica Fwd	AGGCCATCACCGTTTTCAGT
M. domestica Rev	GCGCTGGCCCAGTTGA
D. radicum Fwd	CCATGTTTGTGTGCGGTGTT
D. radicum Rev	GTAGTCTCCAATGCTTCCTGCA
qPCR- *Diap1*	
M. domestica Fwd	GGTTCCGCTGCAACAAAAGT
M. domestica Rev	GGGTGCCTGTTCGTTGGA
D. radicum Fwd	GCCACGCAACAAGAAATAACACCATC
D. radicum Rev	TGACTGAAGAGAACGATGATGA
In vitro synthesis	
M. domestica Fwd	ATAGAATTCGTGGCGGTGGTCTTAAGG
M. domestica Rev	TATAAGCTTGCGACTGCAACCGTTGTATG
D. radicum Fwd	ATAGAATTCCAGTTGTGGTGGTGGTC
D. radicum Rev	TATAAGCTTGCGAGAAGCCTGTTCCG
